# Beliefs Related to Participation in a Large Web-Based Prospective Survey on Diet and Health Among Individuals With a Low Socioeconomic Status: Qualitative Study

**DOI:** 10.2196/13854

**Published:** 2019-12-10

**Authors:** Mélina Côté, Annie Lapointe, Catherine Laramée, Simone Lemieux, Sophie Desroches, Ariane Belanger-Gravel, Benoît Lamarche

**Affiliations:** 1 Institute of Nutrition and Functional Foods Université Laval Québec, QC Canada; 2 School of Nutrition Université Laval Québec, QC Canada; 3 Department of Communication Université Laval Québec, QC Canada

**Keywords:** focus groups, qualitative research, social class, research subject, retention

## Abstract

**Background:**

NutriQuébec is a Web-based prospective study on the relationship between diet and health as well as the impact of food-related health policies in the adult population of Québec, Canada. Recruitment and retention of individuals with a low socioeconomic status (SES) in such a study are known to be challenging, yet critical for achieving representativeness of the entire population.

**Objective:**

This study aimed to identify the behavioral, normative, and control beliefs of individuals with a low SES regarding participation in the NutriQuébec project and to identify their preferences regarding recruitment methods.

**Methods:**

A total of four focus groups were conducted in community centers located in low-income areas of Québec City, Canada. On the basis of the theory of planned behavior, participants’ beliefs associated with attitude, subjective norm, and perceived behavioral control regarding hypothetical participation in the NutriQuébec project were identified. Focus groups were recorded, transcribed, and coded by two analysts.

**Results:**

Participants (16 men and 12 women) were aged between 28 and 72 years, and a majority of the participants had an annual household income of Can $19,999 or less. The main perceived advantages of participating in the NutriQuébec project were contributing to improved collective health and supporting research. The only disadvantage identified was the risk of having to fill out too many questionnaires. Participants could not, in general, identify persons from their entourage who would approve or disapprove their participation in the study. The main facilitators identified were obtaining a brief health assessment and the ability to complete questionnaires in a way that is not Web-based. The main barrier was the lack of internet access. The preferred means of recruitment were through social media, television, and community centers.

**Conclusions:**

These results provide insightful information regarding the best methods and messages to use in order to recruit and retain individuals with a low SES in a population-based prospective study on lifestyle and health on the internet.

## Introduction

### Background

Evaluating and monitoring lifestyle habits, including dietary habits, at the population level is imperative in implementing impactful policies, regulations, and educational programs to improve public health. This requires collection of data from population-based samples that are as representative of the general population as possible. Vulnerable populations, including individuals with a low socioeconomic status (SES), are at the greatest risk of developing chronic diseases as well as adopting poor lifestyle habits such as inadequate dietary habits, sedentary lifestyles, smoking, and alcoholism [1-3]. Such populations are generally underrepresented in health studies [2,4]. Therefore, identifying beliefs such as facilitators and barriers among low SES populations is essential for increasing their participation in health studies and, hence, increasing the generalizability of results from population-based research.

Recruiting individuals with a low SES may represent an even greater challenge in Web-based studies, even if access to the internet and the use of social media have broadened significantly over the recent years [5]. This is of significance because the use of Web-based surveys has become an attractive way of conducting large prospective epidemiological studies at lower costs [6,7]. Perceived barriers to participation among hard-to-reach populations have been reported, including transportation and time constraints [8-10], economic constraints [9], fear of exploitation, and lack of knowledge [9-11]. Yet, the perceived barriers reported in these studies were not documented in the context of a Web-based prospective study and were not conducted according to a validated theoretical framework.

### Theoretical Framework

**Figure 1:**
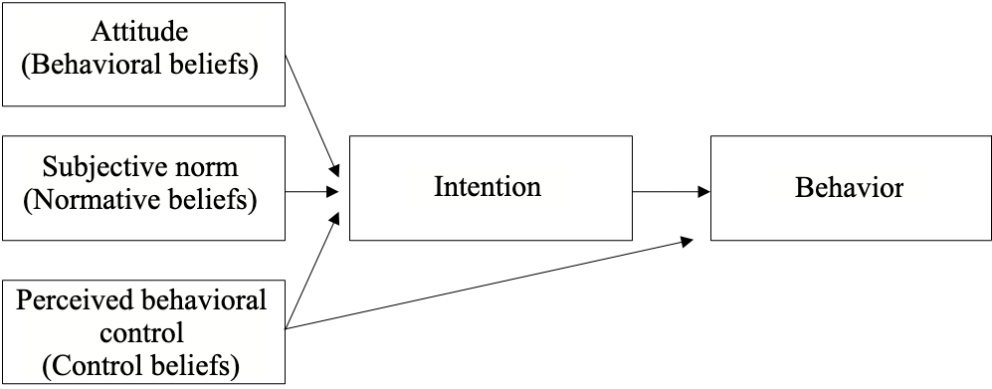
The Ajzen theory of planned behavior.

According to the theory of planned behavior (Figure 1) [12], adoption of a given behavior is determined by one’s intention (or motivation) to perform the behavior. In turn, intention is determined by the attitude toward this behavior; the subjective norm related to this behavior; and perceived behavioral control (PBC), which reflects how one perceives oneself as being capable of performing the behavior. Each of these primary constructs are determined by one’s beliefs. Attitude reflects behavioral beliefs, which are the perceived advantages and disadvantages of performing the behavior. Subjective norms reflect normative beliefs, which are the perceived social pressures of performing or not performing the behavior. PBC reflects control beliefs, which are the perceived facilitators and barriers to one’s ability to perform the behavior. Developing knowledge on beliefs underlying attitudes, subjective norms, and PBC among individuals with a low SES regarding a specific behavior, in this case, the intention to participate in the Web-based NutriQuébec project, will contribute to a better understanding of factors related to the intention to perform the behavior in this population.

### NutriQuébec Project

The NutriQuébec project is a Web-based prospective study aimed at documenting the impact of nutrition-related public health policies and action plans on the diet of the Québec adult population in Canada. More broadly, the NutriQuébec project aims to examine the associations between diet and health outcomes. To this effect, male and female adults (aged ≥18 years) are invited to complete a series of yearly core questionnaires through the Web, assessing dietary habits, physical activity, other lifestyle habits, sociodemographic characteristics, and general health. Time required to complete all questionnaires on a yearly basis is approximately 2 hours (unpublished data). Participants may be invited to complete additional questionnaires on other nutrition-related issues between each yearly core measurements. A brief personalized assessment of dietary habits is returned yearly to each participant as a token of appreciation for their involvement.

To the best of our knowledge, perceptions regarding participation in a Web-based prospective study on nutrition and health have not yet been documented. Thus, the aim of this study was to identify the salient beliefs of individuals with a low SES toward the participation in a prospective Web-based study by using the theory of planned behavior as a framework [12]. We also wanted to identify the methods that were more likely to be successful to recruit and retain individuals with a low SES in such a Web-based study.

## Methods

### Participants and Recruitment

This study used a qualitative approach with focus groups and the theory of planned behavior framework to achieve its objectives. The research team recruited participants through personal contact by going to nonprofit organizations (food banks and community organizations) known to serve populations living in sectors of Québec City (Canada) with a low SES. These sectors are known to have a high rate of unemployment and low level of education. We included adults who were (1) living in a low-income sector, (2) living in the Québec City metropolitan area, and (3) able to speak French. Men and women were invited to participate in a 2-hour focus group to gather opinions about the NutriQuébec project. To accommodate participants, the focus groups were conducted in the community centers visited by the participants. Inclusion criteria were validated on site, and participants were given a date for when the focus group would occur. Participants were called back the day before the focus group to confirm their presence. Participants who completed the focus group received a Can $50 honorarium redeemed in cash and were reimbursed for transportation costs. This study was approved by the Ethics Committee of Laval University (2018-042 A-1/18-05-2018).

### Data Collection

Focus groups were preferred over individual interviews or surveys because they encourage participants to interact with each other, leading to a wider range of salient beliefs, particularly among populations with a low SES who, in general, have lower education levels and lower literacy compared to the rest of the population. A total of four focus groups of six to eight participants each were conducted in three preselected community centers between May 29 and June 12, 2018. Focus groups were audiotaped and videotaped to facilitate verbatim transcription and to document nonverbal behavior when relevant. Before starting the focus group, all participants provided written and informed consent. The participants also completed a questionnaire inquiring about their sociodemographic situation as well as use of the internet. Only a brief description of the soon-to-be-implemented NutriQuébec project was provided to participants to limit biases toward views and perceptions on the potential benefits of participation. A moderator, an assistant moderator, and an observer sat at the table with the participants. The moderator conducted the discussion using a semistructured interview guide (Multimedia Appendix 1), which was developed based on the constructs of the theory of planned behavior [12]. The interview questionnaire was used to identify the beliefs that determine the participants’ attitudes (advantages and disadvantages), subjective norms (approval and disapproval of significant others), and PBC (facilitators and barriers) related to a hypothetical participation in the NutriQuébec project. Participants were also questioned about their concerns regarding the security, use, and sharing of data as well as their preferences regarding recruitment methods. The moderator guide was pretested during the first focus group and needed no modification thereafter. Hence, the first focus group was included in the analysis. The moderator asked the questions in the same order for each of the focus groups to increase comparability of responses and encouraged all participants to express their opinions. The moderator also asked insightful questions and provided prompts when necessary to help move the discussion forward. Responses from the participants in the first focus groups were not used as prompts in subsequent focus groups. Once the participants left, the moderator, assistant moderator, and observer briefly met to comment on the focus group and modal beliefs.

### Data Analysis

The SAS software (SAS Institute, Cary, NC) was used for descriptive analyses. A deductive content analysis [13] of the focus groups was conducted. The verbatim transcriptions were used to code the quotes and categorize the beliefs expressed by the participants according to the constructs of the theory of planned behavior, namely, behavioral beliefs, normative beliefs, and control beliefs or according to their relevance to additional established themes, namely, security and sharing of data and preferred recruitment methods. If a belief was named by several participants in the same group, it was considered as a single belief. Moreover, two analysts (MC and AL) coded the verbatim transcript of each focus group independently using NVivo10 qualitative research software (QSR International Pty Ltd, Melbourne, Australia) before comparing and discussing the identified themes to reach consensus. A third person was available to consult in case of a disagreement. Data saturation, based on informational redundancy, was attained after the fourth focus group [14]. Hence, no additional focus groups were planned.

## Results

### Participants’ Characteristics

A total of 28 participants (16 men and 12 women) aged between 28 and 72 years participated in the study. The majority of participants had an annual household income of Can $19,999 or less, reflecting a low SES. Moreover, three-quarters (21/27, 78%) of the participants had access to the internet at home or elsewhere within their community. Computer skills were self-rated as moderate (6/27, 22%), advanced (10/27, 37%), or expert (3/27, 11%; Table 1).

**Table 1:** Sociodemographic characteristics of the participants from the four focus groups (N=28).

Characteristics		Values	
Age (years), mean (SD)		50 (12)	
**Gender, n (%)**			
	Male		16 (57)
	Female		12 (43)
**Education, n (%)**			
	Primary school		5 (18)
	High school		11 (39)
	College		8 (29)
	University		3 (11)
	I prefer not to answer		1 (4)
**Household income (Can $), n (%)**			
	0-9999		13 (46)
	10,000- 19,999		11 (39)
	20,000- 29,999		3 (11)
	I prefer not to answer		1 (4)
**Computer skills, n (%)^a^**			
	Not at all competent		5 (19)
	Hardly competent		3 (11)
	Moderately and rather competent		6 (22)
	Very competent		10 (37)
	Expert		3 (11)
**Employment situation, n (%)**			
	Full-time worker		0 (0)
	Part-time worker		4 (14)
	Unemployed		6 (21)
	Stay-at-home parent		3 (11)
	Retired		7 (25)
	Unable to work		4 (14)
	Other		3 (11)
	I prefer not to answer		1 (4)
**Internet access, n (%)^a^**			
	Yes		21 (78)
	No		6 (22)

^a^N=27.

### Results of the Analysis of the Focus Groups

All reported beliefs are listed along with quotes to illustrate the key messages and beliefs. The quotes were originally in French and have been translated.

#### Behavioral Beliefs: Perceived Advantages and Disadvantages of Participating in the NutriQuébec Project

Frequently cited advantages of participating in the NutriQuébec project pertained to supporting research, contributing to improving collective health, receiving a brief health assessment, and improving one’s lifestyle habits:

Improve living conditions of people also, not just ourselves. The population in general.

Group 1, participant #3

Other advantages that were mentioned include improving the family’s lifestyle habits, making time for oneself, and acquiring and sharing knowledge.

No clear disadvantages of participating in the NutriQuébec project emerged in most focus groups. In fact, some participants indicated that although they could not identify personal advantages, they did not believe that participating in the project would do any harm or present any disadvantages. The only disadvantage discussed was the risk of having to fill out too many questionnaires.

#### Normative Beliefs: Approval and Disapproval of Significant Others Toward Participation in the NutriQuébec Project

Children, family members, and friends were identified as individuals who would approve participants’ involvement in the NutriQuébec project, and the spouse was identified as an individual who would disapprove. However, half of the focus groups did not identify any individuals who would approve or disapprove their participation in the project. In fact, participants mentioned that they were not influenced by their acquaintances and that their decision to participate in the project would only depend on themselves.

#### Control Beliefs: Facilitators and Barriers to Participating in the NutriQuébec Project

The perceived facilitators were having the possibility to complete the questionnaires using alternative methods to internet and receiving a nonmonetary incentive. The alternative means to internet that were mentioned were having group sessions in predetermined places to complete questionnaires, having the possibility to complete paper questionnaires, receiving questionnaires by regular mail, and filling out questionnaires over the phone. The nonmonetary incentives included a point system, gift cards, a random drawing or contest, and a brief health assessment. Furthermore, participants also acknowledged that they would take part in the project even if no incentive was offered, because the project was important and interesting to them or because they simply did not consider the incentive to be necessary.

Other facilitators discussed were receiving a financial incentive, using simple questions, receiving general information related to food and health, having anonymous questionnaires, having access to a picture of the research team, having a reasonable time frame for completing questionnaires, having tutorials, and providing internet access:

Well, to clearly write “anonymous.” You know, so that...yes, in bold, in capitals, you know so people say “ok, I am nobody when I answer, so I can be myself...”

Group 2, participant #9

The barrier that was most frequently mentioned in the focus groups was the lack of internet access. Other barriers discussed were the absence of a nonmonetary incentive, the lack of time (long-term commitment and questionnaires too long to complete), having limited access to internet, feeling that the study is useless, using complex vocabulary, or the 1-year follow-up leading to forgetting the study.

#### Security and Sharing of Data

Participants were asked to share their thoughts regarding security aspects related to data management. They mentioned that they would not be concerned about data security because of the signed consent form, if the data collected remained anonymous, and if less personal data such as dietary habits were to be collected as compared with more personal data such as presence of a disease:

No, anyways, it’s always about food, there is nothing too personal about that. It doesn’t matter that much.

Group 3, participant #16

Nevertheless, participants also stated that they felt more concerned about data security if health insurance numbers were collected as part of the NutriQuébec project, if the information was provided over the internet, or because they were afraid of data hacking.

Participants expressed no particular concern regarding the sharing of data among researchers as long as the data provided were not modified, because it would allow researchers to have a representative sample of the population of Québec and it would limit the number of research projects (and further solicitation). On the other hand, participants mentioned that they would not be comfortable with data sharing between researchers because it would increase the risk of data leakage.

#### Preferred Recruitment Methods

Participants were also asked to identify the recruitment methods that would most likely be successful in encouraging them to participate in the NutriQuébec project. Channeling recruitment efforts through community groups, social media, television, and brochures were identified as the most promising approaches by the participants. Social media included Facebook, YouTube, and Instagram. Television included news reports as well as other specific channels or television shows. Other recruitment methods mentioned were via posters, newsletters, schools, and hospitals.

## Discussion

### Summary

Our objective in undertaking this qualitative study in a low SES population was to identify the salient beliefs underlying attitudes, subjective norms, and PBC toward participation in a prospective Web-based study on nutrition and health. We also wanted to identify the most promising methods to maximize recruitment of individuals with a low SES in such a project. Results of this study are of relevance not only to the NutriQuébec project but also potentially to any other Web-based surveys that aim to include populations with a low SES.

### Behavioral Beliefs: Perceived Advantages and Disadvantages to Participating in the NutriQuébec Project

Perceived advantages should be part of the key messages used to promote participation in a Web-based project. In our study, contributing to improving collective health, supporting research efforts, receiving a brief health assessment, acquiring and sharing knowledge, improving one’s and the family’s lifestyle habits, and making time for oneself have been identified by these focus groups as key advantages to participating in the NutriQuébec project. However, addressing these advantages is challenging in prospective studies on diet and health such as NutriQuébec because of the risk of interference with the normal trajectory of a cohort. Thus, the means chosen to promote participation in the Web-based project must not encourage nor promote any drastic changes in lifestyle. Yet, data have shown that providing information related to healthy lifestyle habits or a brief health assessment has only trivial long-term impact on a person’s behavior [15].

### Normative Beliefs: Approval and Disapproval of Significant Others Toward Participation in the NutriQuébec Project

The majority of focus groups were unable to identify persons who would approve or disapprove of their participation in the NutriQuébec project. In other words, participants felt that their decision to participate in the project would not be influenced by others. Therefore, acknowledging the subjective norms related to participation among individuals with a low SES in a prospective health study may not be necessary when choosing recruitment and retention methods.

### Control Beliefs: Facilitators and Barriers to Participating in the NutriQuébec Project

The lack of internet access was identified as an important barrier to taking part in the NutriQuébec project. This is not surprising, considering the fact that groups with a low SES show the lowest proportions of internet access in their household [16]. This is consistent with data from Whitaker et al, who have also identified internet access as a barrier to taking part in Web-based studies [17]. In the province of Québec, 90% of households have declared having access to internet, but there is still a gradient related to SES within the population [16]. Indeed, it was estimated that only 59% of low-income families living in the province of Québec had access to internet compared with 98% of moderate-income families [16]. All focus groups suggested the need to allow alternative means to the internet to facilitate participation in the NutriQuébec project, thus highlighting a significant challenge in the context of conducting Web-based epidemiological studies. Nevertheless, the majority of participants (78%) easily had access to the internet at home or elsewhere in their community through local libraries, for example. Although internet access may be perceived as a barrier to participation of individuals with a low SES in Web-based studies, its impact in the coming years will most likely diminish as the internet becomes more broadly available for all segments of the population.

Receiving incentives was mentioned in the focus groups as a facilitator for taking part in the Web-based NutriQuébec project. These data are consistent with results from a previous study indicating that offering an incentive, such as a financial compensation or a gift card, is an effective method for encouraging low-income persons to participate in a study [18]. Hernando et al [19] also reported that the majority of participants would accept a monetary compensation, and this form of incentive was of greater interest among unemployed individuals. Even the use of a modest US $10 monetary incentive was shown to increase the number of returned surveys in a longitudinal study [20]. However, this is not a consistent finding, as Edwards et al [21] have shown that there is no evidence demonstrating that monetary incentives encourage participation in Web-based surveys. It is noteworthy that the monetary incentive was provided via an electronic fund transfer (PayPal), which may have been less accessible for some participants compared with a cash incentive. Providing nonmonetary incentives such as Amazon gift cards, lottery participation, and early grade feedback increased the odds of participants completing Web-based surveys by two-fold [21]. These data are consistent with data from our study in which focus groups expressed a stronger interest in receiving a nonmonetary incentive than a monetary incentive for participating in a lifestyle and health Web-based survey. Receiving a brief health assessment was also mentioned as a facilitator by the focus groups and, thus, is a good example of a promising nonmonetary incentive to facilitate participation in future Web-based studies.

The lack of time was identified as a barrier to possible participation in the NutriQuébec project. This was expressed in the context of the long-term commitment and the time required to complete all questionnaires, which is estimated to be 2 hours. Therefore, the idea of being considerably committed to the study may discourage participation and increase the likelihood of dropping out of the study. This is consistent with data from Hernando et al, who identified time commitment as a key barrier to participation in a longitudinal health study among vulnerable populations, more specifically among migrants [19]. This suggests that retention methods will need to be applied to favor long-term participation. A recent meta-analysis and systematic review on retention strategies in longitudinal cohort studies identified barrier reduction strategies as the most efficient predictor of improved retention [22]. Similarly, Méjean et al reported that reduced participant burden (convenience, simplicity, and not feeling judged) in a Web-based longitudinal study could help reducing attrition [23]. Strategically choosing effective retention strategies rather than increasing the number of strategies was also mentioned as a way to help increase retention rates [22].

All other facilitators and barriers have already been considered in the context of the NutriQuébec project and are applied when possible. For example, the NutriQuébec website uses simple sentences and avoids any complex vocabulary, it offers a thorough explanation of the participant’s implication, and it introduces and describes the research team.

No participant spontaneously identified data security and data sharing among researchers as potential barriers to participating in a lifestyle and health Web-based survey. Concerns regarding security and data sharing were discussed only after the moderator of the focus groups specifically asked the participants their thoughts on these issues. Still, focus groups revealed no particular concern that suggests that only minimal information regarding data security and management needs to be provided to potential participants when undertaking a Web-based study on lifestyle and health.

### Preferred Recruitment Methods

Social media such as Facebook, Instagram, YouTube, and other social media websites were identified in all focus groups as privileged recruitment channels among populations with a low SES. This is consistent with data from studies in other populations where the use of social media contributed to higher enrollment rates compared with other recruitment methods and was successful in recruiting hard-to-reach populations [24]. Indeed, 44% of low-income individuals in Québec connect to social media websites [5]. Using Facebook specifically has also been shown to maximize participation when recruiting hard-to-reach individuals [17,25,26]. In fact, Facebook is not only the most used social media among Canadians but also the most used among low-income families [5]. Studies have shown that using Facebook for recruitment is more cost-effective than traditional recruitment methods [26,27]. Nevertheless, data on the relative efficiency of social media to recruit hard-to-reach populations compared with more traditional recruitment methods remain limited and need further investigation [24].

Recruiting through community centers was identified as another method to reach out to the participants. This is consistent with data from non–Web-based studies that channeled recruitment through community centers to reach out to a vulnerable population. More specifically, advertising a study in community centers was more successful than advertising one in clinical facilities to recruit participants from an ethnic minority [28].

Television was mentioned as an efficient way to promote the study in the focus groups and, hence, recruitment and participation in the NutriQuébec project. This is consistent with a previous study in which the use of advertisement and promotion of a study on television reached the largest number of subjects and was an adequate recruitment method for unemployed individuals, low-income individuals, and individuals with only primary or secondary education compared with other channels [29].

### Limitations

The 28 participants of the focus groups included both men and women and were heterogeneous in terms of computer skills. However, only one participant was aged under 30 years. We do not know the extent to which similar facilitating factors and barriers associated with participating in a Web-based project such as NutriQuébec will be identified by younger individuals with a low SES population. The data from this focus group study also need to be replicated in surveys with larger samples of individuals who will be representative of low SES populations. Finally, although there was evidence of saturation in terms of information yielded from the focus groups, there is a possibility that other facilitators and barriers toward participation in a Web-based study on diet and health would have been discussed if we had included more groups and individuals.

### Conclusions

To our knowledge, this is the first study to document the beliefs and preferred recruitment methods of individuals with a low SES related to their intent to participate in a Web-based prospective study on lifestyle and health. This work is important because populations with a low SES, who are at greater risk of developing chronic diseases, are known to be underrepresented in health studies. Using the theory of planned behavior as a framework, we identified the lack of time, the absence of a nonmonetary incentive, and the lack of internet access as the main barriers. Receiving a brief health assessment (as a nonmonetary incentive) and having alternative means to internet for the completion of questionnaires were mentioned as the main facilitators. Preferred recruitment methods included social media, community centers, and television, and these methods should be considered when recruiting groups with a low SES. Although results from this study are specific to the NutriQuébec project, they are potentially relevant to other Web-based prospective studies aiming to recruit participants with a low SES. However, participants were recruited in the metropolitan area of Québec City and all spoke French. Additional studies in populations with a low SES from other sociocultural backgrounds may yield different results.

## Appendix

Multimedia Appendix 1 Interview guide.

## Abbreviations

PBC: perceived behavioral control
SES: socioeconomic status
