# Supplementary material for: Beliefs Related to Participation in a Large Web-Based Prospective Survey on Diet and Health Among Individuals With a Low Socioeconomic Status: Qualitative Study
Source: JMIR Form Res. 2019 Dec 10;3(4):e13854. doi: 10.2196/13854 (PMC6930510; doi:10.2196/13854)
Supplement: Multimedia Appendix 1 [file formative_v3i4e13854_app1.docx]

NutriQuébec Phase 1: Needs Assessment

**Moderator Guide**

**Presentation of the NutriQuébec project**

Brief presentation of the NutriQuébec project.

*(5 minutes)*

**General questions** (group discussion)

**Before anything, we wish to have your opinion on the future NutriQuébec project. The fact that you are participating in the focus group does NOT imply your participation in the future NutriQuébec project.**

**Core Questionnaires and Complementary Questionnaires**

**Description of the core questionnaires**

As mentioned, the people that will participate in the NutriQuébec project will be invited to complete core questionnaires once per year. The time to complete these questionnaires is estimated at two hours. Note that people will have the possibility of completing these questionnaires on a step-by-step basis, by saving their answers and continuing at another moment, if desired.

1. **If you were a participant in the NutriQuébec project, would you be willing to complete the questionnaires once per year for many years? Why?** (*3 minutes*)

**Description of the complementary questionnaires**

As mentioned, some people participating in the NutriQuébec project will be invited to complete a complementary questionnaire once in a while. The questionnaire will take a maximum of twenty minutes to complete.

1. **If you were a participant in the NutriQuébec project, would you be ready to complete a questionnaire once in a while? Why?** (*3 minutes)*
   1. **For those who would be ready to complete a questionnaire once in a while, how many times per year would you be ready to complete a questionnaire?**

**Completion of Questionnaires**

1. **In cases where you would forget to complete the questionnaires, how would you like us to remind you?** *(3 minutes)*
   1. Probe: means (email, phone, text message), number of reminders
2. **What means could be used to incite you to complete the questionnaires?** *(3 minutes)*
   1. Probe: draw (ex: travel certificate, computer tablet), gifts (ex: movie tickets, grocery gift card), financial compensation, receiving some questionnaire results, etc.
3. **If a financial compensation was not offered, would you accept to participate in the study?**

**Key Questions: Targeted Behaviour (hypothetically)**

Imagine yourself having accepted to participate in the NutriQuébec project, that is, completing questionnaires every year.

**Behavioural Beliefs**

(Attitude- positive or negative consequences)

1. **In our opinion, what would be the advantages to participating in the NutriQuébec project?** *(5 minutes)*
   1. Probe: benefits, interests, good sides
2. **In your opinion, what would be the disadvantages to participating in the NutriQuébec project?** *(5 minutes)*
   1. Probe: negative points, inconveniences, bad sides

**Control Beliefs**

(Perceived behavioural control- prior factors)

1. **In your opinion, what would help you to participate in the NutriQuébec project?** *(5 minutes)*
   1. Probe: what would make it easier, helpful, simpler, more convenient (in terms of means and possibilities). Examples of answers: access to a computer/Internet, health assessment (nutritional advice relating to responses from the questionnaires)
2. **In your opinion, what would prevent you from participating in the NutriQuébec project?** *(5 minutes)*
   1. Probe: barriers, obstacles

**Recruitment**

1. **Which means would be most efficient to recruit you in the NutriQuébec project? If we publicize the NutriQuébec project, how could you hear about us?** *(5 minutes)*
   1. Probe: television, radio, social media (Facebook), newspaper, posters in public places, postal recruitment, etc.
2. **If we were to have a spokesperson to help us popularize the NutriQuébec project, who could it be?** *(5 minutes)*
   1. Probe: celebrity, well-known personality

**BREAK** *(10 minutes)*

Gentle reminder: imagine yourself having accepted to participate in the NutriQuébec project, that is, completing questionnaires every year.

**Personal Data**

**Description of Data Matching**

In the future NutriQuébec project, we could ask participants personal information, like their date of birth, postal code and their health insurance number. These data would be conserved on the study’s highly secured website. These data would not be linked to the participant’s name, which means that the researchers who work with these data could never identify the participants. Of course, these data would only be conserved in the context of research; they would therefore never be sold.

1. **If you were a participant in the NutriQuébec project, what would you think of the fact that we could ask your personal information like:**
   1. **Your date of birth?**
   2. **Your postal code?**
   3. **Would you be willing to provide this information? Why?** *(5 minutes)*

In the future NutriQuébec project, one of the main objectives is to observe the relationships between lifestyle habits and health, for example to see the relationship between what people eat and diseases. Thus, through online questionnaire, questions will be asked to the participants regarding their health status (if they have diseases) and the medication that they take. We would also like to ask participants their health insurance number (RAMQ). This number would allow us to validate the information that will have been provided in the questionnaire. For example, if a participant wrote in a questionnaire that they have diabetes, we will be able to confirm with the RAMQ the diabetes diagnostic and the date of diagnosis. The health insurance number will not allow us to “scour” the medical record of participants. Only the information provided in the questionnaire will be validated. This will allow us to have a more valid and rigorous study.

- 1. **Would you agree to provide your health insurance number? Why?**

**Security, use and sharing of data**

The NutriQuébec project will allow us to collect a big amount of data. These data will be conserved in a secured database. This database will be hosted by the servers at Université Laval and is protected by a password that only the research team will know.

1. **Do you have concerns regarding the security of the data that will be collected using questionnaires on the web?** *(5 minutes)*
   1. **Could your concerns regarding the security of the data lead you to not want to participate in the NutriQuébec project?**
   2. **What could we do to reduce your worries regarding the security of data?**

In the NutriQuébec project, the data will be confidential. The name of participants will be replaced by a code for all research data holding personal information. Many researchers will be able to use these data.

1. **Do you have concerns regarding the use and sharing of data between researchers, knowing that they will not have access to your personal data, like you name and your health insurance number?** *(5 minutes)*
   1. **Could your concerns regarding the use and sharing of data between researchers lead you to not want to participate in the NutriQuébec project?**
   2. **What could we do to calm your worries regarding the use and sharing of data between researchers?**

**Clinico-biological data**

In the future NutriQuébec project, we could invite some participants to come to the research center to do clinical tests (weight and height measurement, blood pressure, blood test, etc.). These data could allow us to study the relationship between what people eat and their weight for example.

1. **If you were a participant in the NutriQuébec project, would you accept to do clinical tests (weight and height measurement, blood pressure, blood test, etc.)? Why?** *(3 minutes)*
2. **What means could be used to incite you to do the clinical tests?** *(5 minutes)*
   1. Probe: draw (ex: travel certificate, computer tablet), gifts (ex: movie tickets, grocery gift card), financial compensation, receiving some questionnaire results, etc.

**Normative beliefs**

1. **In your environment (work, home, leisure), which important persons to you (or group of people) would have an influence on your participation in studies?** *(3 minutes)*
   1. In your immediate environment
   2. In your broader environment
2. **Among the persons (groups or individuals) that are important to you, which would be the ones that would approve your participation in the NutriQuébec project? For which reasons?** *(3 minutes)*
   1. Probe: examples (during the completion of questionnaire, during a discussion at supper, etc.)
3. **Among the persons (groups or individuals) that are important to you, which would be the ones that would disapprove your participation in the NutriQuébec project? For which reasons?** *(3 minutes)*
   1. Probe: subtle way of disapproving: non-verbal.

**Final Question:**

1. **Do you have other comments to add regarding the NutriQuébec project?** *(2 minutes)*

*Thank you for your participation!*
